# Connectivity-Based Brain Network Supports Restricted and Repetitive Behaviors in Autism Spectrum Disorder Across Development

**DOI:** 10.3389/fpsyt.2022.874090

**Published:** 2022-03-25

**Authors:** Anyi Zhang, Lin Liu, Suhua Chang, Le Shi, Peng Li, Jie Shi, Lin Lu, Yanping Bao, Jiajia Liu

**Affiliations:** ^1^Peking University Sixth Hospital, Peking University Institute of Mental Health, NHC Key Laboratory of Mental Health (Peking University), National Clinical Research Center for Mental Disorders (Peking University Sixth Hospital), Peking University, Beijing, China; ^2^National Institute on Drug Dependence and Beijing Key Laboratory on Drug Dependence Research, Peking University, Beijing, China; ^3^Peking-Tsinghua Centre for Life Sciences and PKU-IDG/McGovern Institute for Brain Research, Peking University, Beijing, China; ^4^School of Public Health, Peking University, Beijing, China; ^5^School of Nursing, Peking University, Beijing, China

**Keywords:** autism spectrum disorder, brain networks, network-based statistic, restricted and repetitive behaviors, mediation analysis

## Abstract

**Introduction:**

Autism spectrum disorder (ASD) is a lifelong condition. Autistic symptoms can persist into adulthood. Studies have reported that autistic symptoms generally improved in adulthood, especially restricted and repetitive behaviors and interests (RRBIs). We explored brain networks that are related to differences in RRBIs in individuals with ASDs among different ages.

**Methods:**

We enrolled 147 ASD patients from the Autism Brain Imaging Data Exchange II (ABIDEII) database. The participants were divided into four age groups: children (6–9 years old), younger adolescents (10–14 years old), older adolescents (15–19 years old), and adults (≥20 years old). RRBIs were evaluated using the Repetitive Behaviors Scale-Revised 6. We first explored differences in RRBIs between age groups using the Kruskal–Wallis test. Associations between improvements in RRBIs and age were analyzed using a general linear model. We then analyzed RRBIs associated functional connectivity (FC) links using the network-based statistic method by adjusting covariates. The association of the identified FC with age group, and mediation function of the FC on the association of age-group and RRBI were further analyzed.

**Results:**

Most subtypes of RRBIs improved with age, especially stereotyped behaviors, ritualistic behaviors, and restricted behaviors (*p* = 0.012, 0.014, and 0.012, respectively). Results showed that 12 FC links were closely related to overall RRBIs, 17 FC links were related to stereotyped behaviors. Among the identified 29 FC links, 15 were negatively related to age-groups. The mostly reported core brain regions included superior occipital gyrus, insula, rolandic operculum, angular, caudate, and cingulum. The decrease in FC between the left superior occipital lobe and right angular (effect = −0.125 and −0.693, respectively) and between the left insula and left caudate (effect = −0.116 and −0.664, respectively) might contribute to improvements in multiple RRBIs with age.

**Conclusion:**

We identified improvements in RRBIs with age in ASD patients, especially stereotyped behaviors, ritualistic behaviors, and restricted behaviors. The decrease in FC between left superior occipital lobe and right angular and between left insula and left caudate might contribute to these improvements. Our findings improve our understanding of the pathogenesis of RRBIs and suggest potential intervention targets to improve prognosis in adulthood.

## Introduction

Autism spectrum disorder (ASD) comprises a set of lifelong neurodevelopmental disorders that develop in early childhood, characterized by such core symptoms as social deficits and restricted and repetitive behaviors and interests [RRBIs; (1)]. The prevalence of ASD is estimated to be 1 in 59, which has dramatically increased in recent decades and caused numerous public health and social burdens worldwide (2). The onset of ASD symptoms among toddlers appears around 18–24 months of age, and a reliable diagnosis is typically made at 3–4 years of age (3). The early onset of deficits in joint attention and verbal or non-verbal communication contribute to early identification (1). Early screening and intervention usually indicate a better outcome in adulthood. Importantly, ASD is a lifelong condition. Its clinical symptoms might persist into adulthood (4). The developmental trajectory of autistic symptoms is largely unknown. Although autism was considered a “devastating condition,” subsequent studies reported that autistic symptoms generally improved in adulthood, especially RRBIs that might decrease over time (5–7). According to the finding in typically developing children, the higher-order RRBIs decreased later than lower-order RRBIs (8). Generally, the improvement of RRBIs in autistic children was similar to that in typically developing children, but happened later (5).

There are several subtypes of RRBIs, including stereotyped or repetitive motor movements, insistence on sameness, highly restricted and fixated interests, and hyper- or hyporeactivity to sensory input, according to the *Diagnostic and Statistical Manual of Mental Disorders*, 5th edition (1). As a core symptom of ASD, RRBIs might first be found at 2 years of age, expressed as repetitively playing with specific toys or abnormal sensory interests, and gradually increased with age before 6 years old (9). The severity of repetitive motor movements and rigid routines is highest at preschool age and then decreases during school age and adolescence (10).

Children's brains are rapidly growing and developing systems. Recent studies identified atypical developmental trajectories of brain connectivity in individuals with ASD. These alterations might contribute to different autistic symptoms throughout life (11). Brain connectomics analyses in ASD patients identified an atypical age-associated trajectory of connectivity in the default mode network [DMN; (12)]. Studies of developmental changes in large-scale brain networks indicated that within and between network connectivity were not uniform at different ages (13). A previous review proposed a developmental model to explain age-related connectivity in ASD patients, including hyperconnectivity in childhood and hypoconnectivity in adulthood (14). These findings indicate atypical developmental trajectories in ASD patients over time, which might contribute to different clinical characteristics at different developmental stages.

According to studies of brain mechanisms that underlie RRBIs, structural abnormalities of the striatum might be involved in RRBIs in autism (15). Little is known about connectivity-based brain characteristics of age-related developmental trends of RRBIs. The present study explored brain network organization that is related to RRBIs in ASD patients of different ages. We expected to generate a comprehensive model of age-related brain network features that support RRBIs in ASD. Our findings will help understand the pathogenesis of age-related changes in RRBIs and provide crucial clues to aid the development of new treatments and interventions at early stages of ASD and improve prognosis in adulthood.

## Materials and Methods

### Participants

Participants were recruited from the Autism Brain Imaging Data Exchange II (ABIDEII; http://fcon_1000.projects.nitrc.org/indi/abide/). There were 1,144 subjects who were 5-64 years old in the ABIDEII database, including 521 individuals with ASD [diagnosed by Autism Diagnostic Observation Scale (ADOS) and/or Autism Diagnostic Interview-Revised (ADI-R)] and 593 controls (16). Participants were included if they (1) were ≥ 6 years old, (2) were diagnosed with ASD, (3) had complete data from the Repetitive Behaviors Scale-Revised 6 (RBSR-6), and (4) were right-handed. The exclusion criteria were (1) missing data on age, sex, full intelligence quotient (FIQ), and current medication status, (2) no functional or structural imaging data, and (3) large head motions (3.0 mm translation or 3.0° rotation). The final sample included 147 ASD patients (123 males and 24 females) from nine sites: Barber National Institute (BNI), Institut Pasteur (IP), Kennedy Krieger Institute (KKI), Katholieke Universiteit Leuven (KUL), New York University (NYU), San Diego State University (SDSU), Trinity College Dublin (TCD), and University College Dublin (UCD). The ASD patients were classified into four groups according to the World Health Organization classification: (1) children (6–10 years old; 31 subjects), (2) younger adolescents (11–14 years old; 52 subjects), (3) older adolescents (15-19 years old; 33 subjects), and (4) adults (≥20 years old; 31 subjects) (17).

### Ethics

The data in the present study was extracted from an open online database (http://fcon_1000.projects.sitrc.org/indi/abide/). All participants and their caregivers signed informed consent forms at each site.

### Measures

#### RBSR-6

The RBSR-6 is a widely used questionnaire that evaluates RRBIs. It includes 43 items that are grouped into six RRBI subtypes: stereotyped behaviors, self-injurious behaviors, compulsive behaviors, ritualistic behaviors, sameness behaviors, and restricted behaviors. Every item is ranked on a 4-point Likert-type scale, from 0 (do not occur) to 3 (very serious) (18). The assessment of RRBIs using the RBSR-6 is usually based on observations by parents, caregivers, teachers, or others who know the patient. This questionnaire has excellent reliability and validity (19).

#### Covariates

We further extracted age, sex, handedness, FIQ, eye-status during scan, and current medication status from the online database (http://fcon_1000.projects.sitrc.org/indi/abide/). Handedness was grouped into three categories: right-handed, left-handed, and mixed-handed. Only right-handed patients were enrolled in the study. Measurements of FIQ were different across sites: Kaufman Brief Intelligence Test at BNI, Wechsler Intelligence Scale for Children at IP and KKI, Wechsler Adult Intelligence Scale at KUL, and Wechsler Abbreviated Scale of Intelligence at NYU, SDSU, TCD, and UCD. The results of the different measurements were comparable (20, 21). Eye-status during scan was grouped into two categories: open and closed. Medication status about whether the participants currently took medications within 3 months before the scan was also extracted from the database. These data were controlled as covariates in the analysis.

### Brain Imaging Processing

Imaging data were preprocessed using the Data Processing Assistant for Resting-State Functional Magnetic Resonance Imaging (DPARSF) in MATLAB (22). The first 10 volumes for each subject were removed for magnetization equilibrium. The remaining volumes were slice-time corrected, head-motion realigned, co-registered to structural scans, normalized to 3 × 3 × 3 mm^3^ voxels with a bounding box of [−90 −126 −72; 90 90 108], smoothed with a Gaussian kernel with a full-width half maximum of 6 mm, and bandpass filtered (0.01–0.1 Hz). The detailed scan parameters were presented in Supplementary Table 1.

After preprocessing, we constructed a whole-brain functional connectivity (FC) network for each participant in DPARSF. The whole brain was segmented into 116 brain regions based on the Anatomical Automatic Labeling atlas, and regional signals were extracted by averaging blood oxygen level-dependent signals of every voxel in the brain region. We then calculated FC between every two brain regions using the Pearson correlation between regional signals (116 × 116 brain regions with 13,456 edges for all of them).

### Statistical Analysis

Demographic characteristics are presented as percentages for categorical variables and means, standard deviations (SDs) or medians, and interquartile ranges (IQRs) for continuous variables. Comparisons of RRBIs in the different age groups were conducted using the Kruskal–Wallis test. We additionally compared the subscales evaluating restricted/repetitive behaviors in ADOS and ADI-R among different age-group using the Kruskal–Wallis test, and results were presented in Supplementary Table 2. Associations between age groups and RRBIs were further investigated using a general linear model (GLM) after adjusting for sex, age, FIQ, and current medication status. These analyses were performed using Statistical Package for the Social Sciences (SPSS) 23.0 software.

The network-based statistic (NBS) was applied to investigate functional networks that were associated with RRBIs in ASD patients after controlling for age, sex, FIQ, eye-status during rest scan, and medication status (23). The NBS process was conducted as the following: (1) conduct a linear regression model to explore every edge associated with RRBIs after controlling covariates, and further perform *t*-tests for those associated components, (2) identify connected components that were suprathreshold (threshold = 3.0) links, and (3) compute the family-wise error-corrected *p*-value for each connected components using 5,000 random permutations. Correlations between RRBIs-related FC links and age groups were then investigated using Pearson correlation analysis by controlling for sex, age, FIQ, eye-status during scan, and medication status.

Shared FC links that were related to the RRBI subscales and age groups might simultaneously contribute to developmental changes in RRBIs with age in ASD patients. We conducted a mediation analysis to explore whether shared FC links might mediate the relationship between developmental stages and RRBIs. We used Model 4 in PROCESS, and *N* = 10,000 was used for the bootstrap. The level of significance was set at *p* < 0.05 (two-tailed).

## Results

### Demographic Characteristics

Demographic characteristics are presented in Table 1. Included participants were between 6 and 64 years of age, and the median age was 17 years old. Most participants in our study were male (83.67%). The FIQ of the subjects ranged from 66 to 146. Most patients had high-functioning autism (IQ > 70).

**Table 1:** Demographic characteristics (*n* = 147).

**Item**	**Description**
Male [*n* (%)]	123 (83.67%)
Age (years) (median, IQR)	17.37 ± 11.57
Age-group [*n* (%)]	
Children	31 (21.09%)
Younger adolescents	52 (35.37%)
Older adolescents	33 (22.45%)
Adults	31 (21.09%)
FIQ (mean ± SD)	104.56 ± 15.00
Currently taking medication [*n* (%)]	39 (26.5%)
RBSR-6 total raw score (median, IQR)	20.00, 22.00
Stereotyped behaviors	3.00, 4.00
Self-injurious behaviors	0.00, 2.00
Compulsive behaviors	2.00, 4.00
Ritualistic behaviors	4.00, 5.00
Sameness behaviors	6.00, 7.00
Restricted behaviors	3.00, 4.00
Eyes opened during scanning [*n* (%)]	102 (69.39%)

*IQR, interquartile range; SD, standard deviation; RBSR-6, Repetitive Behaviors Scale-Revised 6*.

### RRBIs at Different Developmental Stages

**Figure 1:**
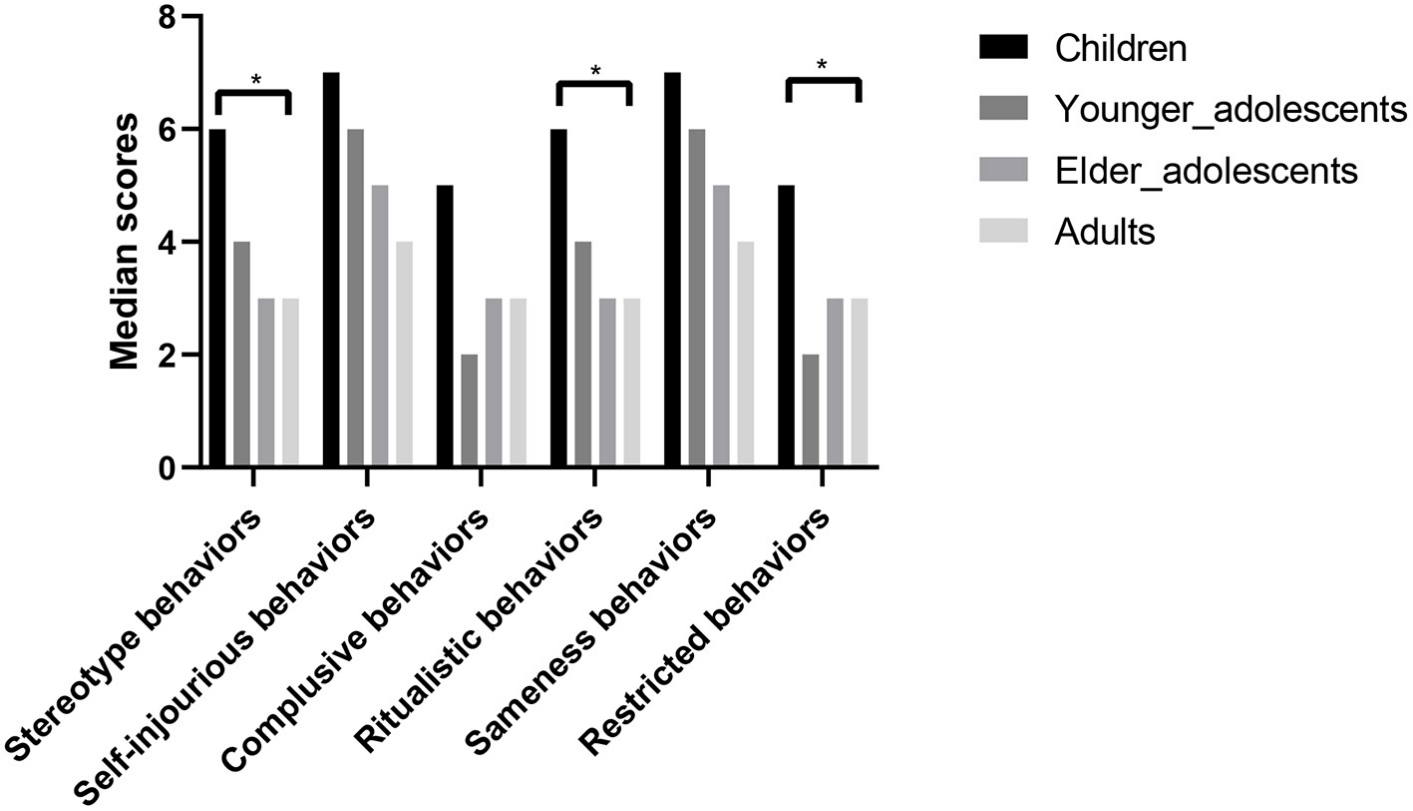
RRBI subtype scores at different developmental stages (*n* = 147). Median scores are shown for six subtypes of RRBIs (stereotyped behaviors, self-injurious behaviors, compulsive behaviors, ritualistic behaviors, sameness behaviors, and restricted behaviors) in four age groups: children (6–9 years old), younger adolescents (10–14 years old), older adolescents (15–19 years old), and adults (≥20 years old). Comparisons of median scores among different age groups were performed using the Kruskal–Wallis test. Children had the highest scores, and then their scores for every subtype gradually decreased with age. The decreases in stereotyped behaviors, ritualistic behaviors, and restricted behaviors were statistically significant. *p < 0.05.

Figure 1 shows the severity of RRBI subtypes in the different age groups. The trajectories of each subtype showed similar developmental patterns, in which children had the highest scores, which then gradually decreased with age. According to the results of the Kruskal–Wallis test, improvements in stereotyped behaviors, ritualistic behaviors, and restricted behaviors were statistically significant (*p* = 0.012, 0.014, and 0.012, respectively). The *post-hoc* tests showed significant differences between children and the older age groups. Detailed RRBI subtype scores in the different age groups are presented in Table 2.

**Table 2:** Description of RRBIs in different age groups (*n* = 147).

**Item**	**(1) Children** **(*n* = 31)**	**(2) Younger adolescents** **(*n* = 52)**	**(3) Older adolescents** **(*n* = 33)**	**(4) Adults** **(*n* = 31)**	***H***	***p***	***p*** **in** ***post-hoc*** **test**					
							**(2)–(1)**	**(3)–(1)**	**(4)–(1)**	**(3)–(2)**	**(4)–(2)**	**(4)–(3)**
RBSR-6 total raw score	32.77, 21.54	23.40, 17.69	22.42, 16.68	20.48, 14.48	7.89	0.048	0.020	0.036	0.014	0.967	0.630	0.693
Stereotyped behaviors	4.81, 3.46	3.31, 2.84	3.42, 3.24	2.32, 1.99	10.75	0.013	0.026	0.060	0.001	0.918	0.159	0.176
Self-injurious behaviors	2.23, 3.47	1.92, 2.75	1.52, 2.69	0.94, 1.71	5.22	0.157	NA	NA	NA	NA	NA	NA
Compulsive behaviors	5.19, 4.98	3.42, 3.76	3.12, 4.20	3.65, 3.77	7.08	0.070	NA	NA	NA	NA	NA	NA
Ritualistic behaviors	6.77, 4.40	4.94, 3.90	3.97, 3.89	3.87, 3.26	10.28	0.016	0.054	0.004	0.008	0.209	0.288	0.881
Sameness behaviors	9.03, 7.18	6.79, 6.44	7.15, 6.28	6.94, 6.04	4.56	0.207	NS.	NS.	NS.	NS.	NS.	NS.
Restricted behaviors	4.74, 3.30	2.98, 2.51	3.24, 2.67	2.77, 2.43	9.20	0.027	0.006	0.066	0.011	0.498	0.888	0.471

*RRBIs, restricted and repetitive behaviors and interests; IQR, interquartile range; RBSR-6, Repetitive Behaviors Scale-Revised 6; NA, not applicable. Post-hoc tests were conducted to explore differences between age groups. In the post-hoc test, (1) refers to children, (2) refers to younger adolescents, (3) refers to older adolescents, and (4) refers to adults*.

Associations between the severity of RRBIs and age groups are shown in Table 3. Because this was an observational study, GLM analysis showed that age was negatively associated with the severity of stereotyped behaviors, ritualistic behaviors, restricted behaviors, and overall RRBIs. Associations with other RRBI subtypes were not statistically significant (Table 3).

**Table 3:** Association between age and severity of RRBIs based on GLM (*n* = 147).

**Age group**	**Total raw score**	**Stereotyped behaviors**	**Self-injurious behaviors**	**Compulsive behaviors**	**Ritualistic behaviors**	**Sameness behaviors**	**Restricted behaviors**
Children (reference)	1	1	1	1	1	1	1
Younger adolescents	−9.37* (−17.16, −1.58)	−1.50* (−2.78, −0.22)	−0.30 (−1.50, 0.90)	−1.77 (−3.59, 0.05)	−1.83* (−3.54, −0.13)	−2.24 (−5.09, 0.60)	−1.76* (−2.95, −0.57)
Older adolescents	−10.35* (−18.93, −1.77)	−1.38 (−2.80, 0.03)	−0.71 (−2.03, 0.61)	−2.07* (−4.08, −0.07)	−2.80* (−4.68, −0.93)	−1.88 (−5.02, 1.26)	−1.50* (−2.81, −0.19)
Adults	−12.29* (−21.01, −3.57)	−2.48* (−3.92, −1.05)	−1.29 (−2.63, 0.05)	−1.55 (−3.59, 0.49)	−2.90* (−4.81, −1.00)	−2.10 (−5.28, 1.09)	−1.97* (−2.95, −0.57)

The statistics show β and 95% confidence interval. RRBIs, restricted and repetitive behaviors and interests. The models adjusted for sex, age, FIQ, eye status during scan and current medication status. * p < 0.05.

### Functional Connectivity Links Associated With RRBI Subtypes

**Figure 2:**
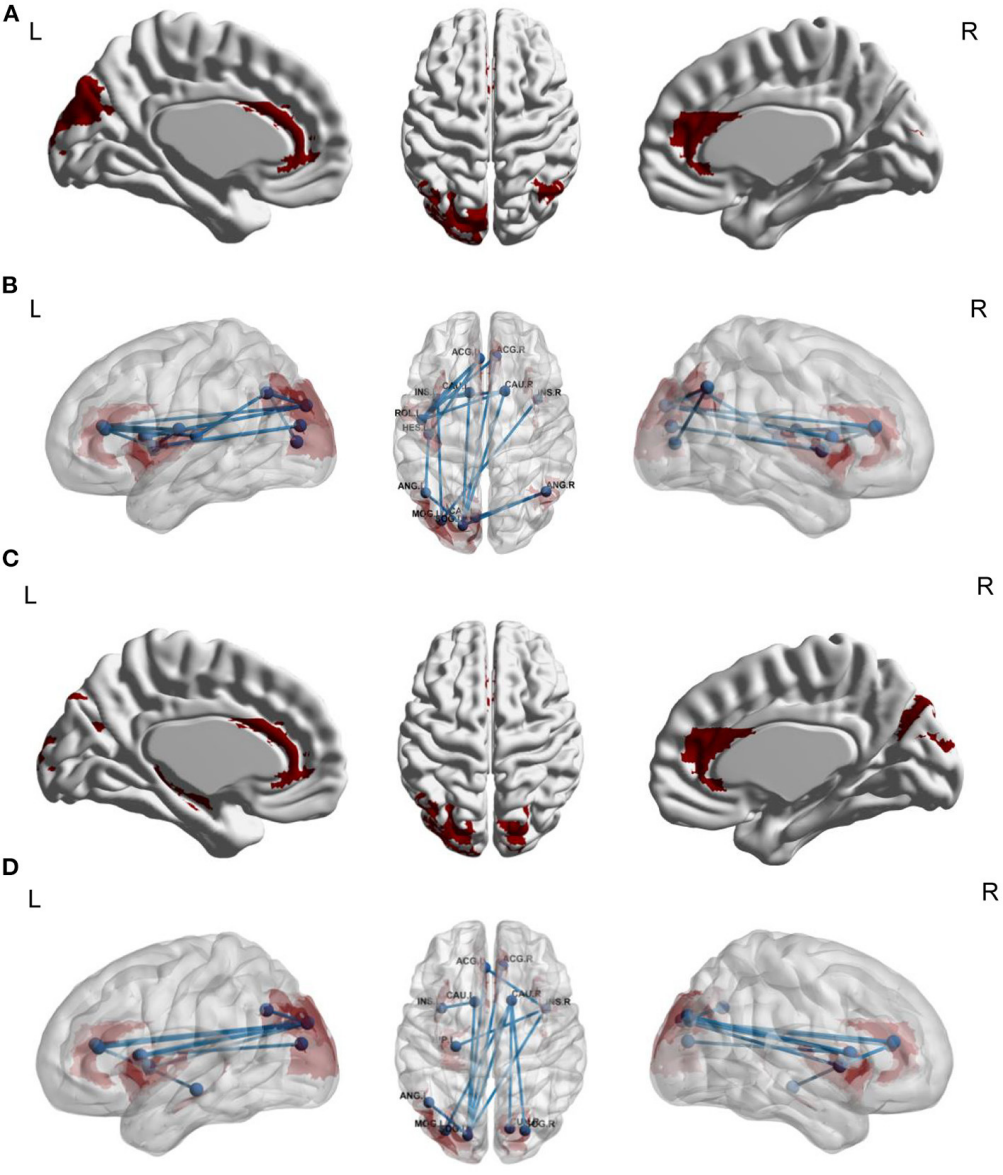
Functional connectivity links associated with stereotyped behaviors **(A,B)** and overall RRBIs **(C,D)** (*n* = 147). **(A,B)** Network-based statistic results for stereotyped behaviors in ASD patients. The underlying functional network included 17 edges between 13 brain regions. **(C,D)** Network-based statistic results for overall RRBIs. The correlated connectivity network included 12 edges between 12 brain regions. In the NBS analysis, we adjusted several covariates, including sex, age, FIQ, eye-status during scan, and medication status. ACG_L, left anterior cingulate gyrus; ACG_R, right anterior cingulated gyrus; CAU_L, left caudate; CAU_R, right caudate; INS_L, left insular; INS_R, right insular; ROL_L, left rolandic operculum; HES_L, left heschl's gyrus; ANG_L, left angular; ANG_R, right angular; MOG_L, left middle occipital gyrus; SOG_L, left superior occipital gyrus; CAL_L, left calcarine; ACG_L, left anterior cingulate gyrus; ACG_R, right anterior cingulate gyrus; HIP_L, left hippocampus; CUN_R, right cuneus; SOG_R, right superior occipital gyrus.

We explored FC links that were associated with stereotyped behaviors, ritualistic behaviors, restricted behaviors, and overall RRBIs. For stereotyped behaviors, the underlying functional network included 17 edges between 13 brain regions (Table 4, Figures 2A,B). We did not find FC links that were associated with ritualistic behaviors or restricted behaviors. For overall RRBIs, the correlated connectivity network included 12 edges between 12 brain regions (Table 4, Figures 2C,D). We then extracted the 17 and 12 FC links for each individual, and the association between FC links and age were also calculated. It was found that 15 out of the 29 FC links were negatively related to age, meaning that FC between these 13 brain regions decreased with age (Table 4).

**Table 4:** Functional connectivity associated with stereotyped behaviors and overall RRBIs and correlation between FC and age (*n* = 147).

**Dependent variable**	**Brain region 1**	**Brain region 2**	**Connection strength**	**Correlation with age**
Stereotyped behaviors	Occipital_sup_L	Angular_R	3.39	−0.35**
	Occipital_sup_L	Caudate_L	3.18	−0.16
	Occipital_sup_L	Cingulum_ant_R	3.04	−0.20*
	Occipital_sup_L	Caudata_R	3.60	−0.16
	Occipital_sup_L	Angular_L	3.02	−0.35**
	Cuneus_L	Angular_R	3.41	−0.38**
	Insula_R	Occipital_mid_L	3.08	−0.13
	Insula_L	Occipital_mid_L	3.04	−0.13
	Insula_L	Caudate_L	3.18	−0.189*
	Insula_L	Caudate_R	3.15	−0.21*
	Insula_L	Cingulum_ant_L	3.22	−0.26*
	Insula_L	Rolandic_oper_L	3.59	−0.20*
	Angular_L	Heschl_L	3.10	−0.26*
	Cingulum_ant_L	Heschl_L	3.03	−0.23*
	Rolandic_oper_L	Caudate_R	3.27	−0.23*
	Rolandic_oper_L	Cingulum_ant_L	3.05	−0.29**
	Rolandic_oper_L	Cingulum_ant_R	3.16	−0.31*
Overall RRBIs	Occipital_sup_R	Caudate_R	3.31	−0.14
	Cuneus_R	Caudate_R	3.00	−0.15
	Occipital_sup_L	Angular_R	3.10	−0.35**
	Occipital_sup_L	Insula_R	3.36	−0.07
	Occipital_sup_L	Caudate_R	4.02	−0.16
	Occipital_sup_L	Caudate_L	3.50	−0.16
	Occipital_sup_L	Cingulum_ant_R	3.43	−0.20*
	Occipital_sup_L	Cingulum_ant_L	3.12	−0.19*
	Insula_R	Hippocampus_L	3.01	−0.25*
	Insula_R	Cingulum_ant_L	3.10	−0.25*
	Occipital_mid_L	Caudate_R	3.30	−0.18
	Insula_L	Caudate_L	3.03	−0.19*

RRBIs, restricted and repetitive behaviors and interests; NBS, network-based statistic; R, right hemicerebrum; L, left hemicerebrum. The models adjusted for sex, age, FIQ, eye status during scan and current medication status. ^*^p < 0.05, ^**^p < 0.001.

### FC Links Mediated the Association Between Age and RRBIs

As shown in Table 5, FC between left superior occipital lobe and right angular, left superior occipital lobe and left angular, left cuneus and right angular, left insula and left caudate, left caudate and right caudate mediated improvements in stereotyped behaviors with age. Interestingly, all these forementioned FC links negatively mediated the difference of stereotyped behaviors and overall RRBIs among age groups, which meant that the FC between these brain regions were decreased with age, and the decreased connectivities might further contribute to the improvement of different domains of BBRIs in ASD patients. Moreover, FC between left superior occipital lobe and right angular, left insula and left caudate were related to improvements in total RRBIs with age simultaneously, which might indicate that FC links between the left superior occipital lobe and right angular, left insula and left caudate might contribute to developmental changes in multiple RRBIs in patients with ASD.

**Table 5:** Functional connectivity that mediated associations between age and stereotyped behaviors/overall RRBIs (*n* = 147).

**X**	**M**	**Y**	**Mediation effect**			
			**Effect**	**SE**	**LLCI**	**ULCI**
Age groups	Occipital_sup_L+Angular_R	Stereotyped behaviors	−0.125	0.074	−0.292	−0.003
	Occipital_sup_L+Angular_L		−0.110	0.069	−0.270	−0.001
	Cuneus_L+ Angular_R		−0.136	0.077	−0.312	−0.012
	Insula_L+Caudate_L		−0.116	0.075	−0.288	−0.003
	Insula_L+Caudate_R		−0.118	0.077	−0.295	−0.005
Age groups	Occipital_sup_L+Angular_R	Overall RRBIs	−0.693	0.408	−1.605	−0.027
	Insula_L+Caudate_L		−0.664	0.435	−1.672	−0.015

*RRBIs, restricted and repetitive behaviors and interests; X, independent variables; M, mediators; Y, dependent variables; SE, standard error; LLCI, lower level of 95% confidence interval; ULCI, upper level of 95% confidence interval; R, right hemicerebrum; L, left hemicerebrum*.

## Discussion

We identified improvements in RRBIs with age, especially stereotyped behaviors, ritualistic behaviors, and restricted behaviors. Furthermore, FC between the left superior occipital lobe and right angular and between the left insula and left caudate, which are responsible for visual integration, motor function, and sensorimotor process, might contribute to developmental changes in multiple RRBIs in patients with ASD. To our knowledge, this was the first study to analyze neuroimaging-based features that support trajectories of RRBIs in ASD patients. These core brain regions might have implications for future intervention targets.

Our results indicated that the RRBI subscales had different improvement trajectories. Stereotyped behaviors, ritualistic behaviors, and restricted behaviors improved greatly after childhood. These finding corroborated previous studies that reported a broad range of trajectories of RRBIs in ASD patients (8–10, 24). For children, RRBIs are relatively stable between 2 and 7 years of age (9). Richler et al. reported discrepant trajectories between different subtypes of RRBIs, in which repetitive sensory motor behaviors (i.e., repetitive motor movements) and the repetitive usage of objects would increase while the insistence of sameness (i.e., adherence to daily routines) would decrease during a 7-year follow-up period (10). Moreover, RRBIs also manifest in typically developing children, and developmental changes of RRBIs in healthy toddlers suggest that higher-order RRBIs (e.g., insistence on sameness) decrease later than lower-order RRBIs (e.g., stereotype behaviors; (8). In summary, the trajectory of autistic symptoms in ASD patients suggests stable improvements in RRBIs over time. We were able to measure age-related improvements in stereotyped behaviors, ritualistic behaviors, and restricted behaviors in the present study. The present GLM analysis indicated that compulsive behaviors greatly improved after younger adolescence, which occurred slightly later than improvements in stereotyped behaviors, which was consistent with a previous study (8).

Atypical neurodevelopment has been previously reported to contribute to autism-related symptoms. Previous brain development studies mostly focused on cortical volume, gray matter thickness, or white matter volume (25). We found that significant reductions of FC among brain regions in the temporal lobe, occipital lobe, and cingulate might contribute to the trajectory of RRBIs over time, especially stereotyped behaviors and overall RRBIs (evaluated by the RBSR-6). Previous studies that examined associations between cortical thickness and RRBI severity over time found that gray matter thickness of the orbital frontal cortex and middle frontal cortex correlated with self-injurious behaviors in adolescent patients (24). In the present study, core brain regions that were responsible for stereotyped behaviors included the superior occipital gyrus, insula, rolandic operculum, angular, caudate, and cingulum. Importantly, these FC links declined with age at the same time. In ASD patients, neurobiology studies found that the most reported brain regions that were responsible for RRBIs were located in corticostriatal circuits, including the orbitofrontal cortex, anterior cingulate cortex, caudate, putamen, pallidum. These brain regions that are associated with goal-directed behaviors, alterations of growth rate, abnormalities in cortical thickness or volume, decreases or increases in FC between brain regions, and changes in neuro-metabolism might contribute to atypical behaviors, including RRBIs (15, 26, 27). The present study supported the implication of these brain regions. We also found that stereotyped behaviors were related to alterations of FC between the superior occipital gyrus, angular, insula, rolandic operculum, and Heschl gyrus, which are mostly related to sensory processing and integration.

The superior occipital gyrus is important for visual processing and fluid reasoning (27, 28). Nebel et al. found that atypical FC between the superior occipital gyrus and motor cortex might contribute to visual-motor disfunction in school-age ASD patients (29). Thus, alterations of FC of the superior occipital gyrus might have been responsible for sensorial stereotyped behaviors that are related to visual processes in the present study.

The angular gyrus is in the parietal lobe and part of the DMN (30). Dysfunction of the angular gyrus has been reported to be involved in pathophysiological social cognition and social deficits in ASD patients (31). The angular gyrus is also an important hub that converts multisensorial messages and manages multicognition processes (32). With regard to RRBIs, abnormal FC of the angular gyrus might be an attempt to correct atypical FC in corticostriatal circuits (15, 30).

The insular cortex is located at the base of the lateral sulcus, consisting of three subregions that are based on distinct functions: dorsal anterior insular (involved in cognitive control), ventral insula (involved in emotion and affective processing), and posterior insula (involved in sensorimotor processing) (33, 34). Structural and functional abnormalities of the insula have been reported to contribute to social and non-social cognitive impairments in ASD patients (34). The volume of the caudate, the density of caudate nuclei, and atypical structural or FC between frontal-striatal circuits have been reported to underlie RRBIs (15, 35, 36).

The operculum covers the insula, which is related to emotional processing and facial expressions (37, 38). Zhou et al. found that RRBIs in ASD patients were closely related to a decrease in FC between the rolandic operculum and anterior cingulum cortex (39). The rolandic operculum is also an important part of the sensorimotor network, and a decrease in connectivity of the rolandic operculum might contribute to deficits in sensory processing and motor function. Moreover, ASD patients were suggested to engage in stereotyped or compulsive behaviors to produce sensory self-stimulation, reflecting a sensory processing deficit (39, 40).

The Heschl gyrus is part of the temporal lobe, located mainly at the primary auditory cortex (41). In ASD patients, bottom-up sensorimotor network dysfunction is common. The Heschl gyrus plays an important role in auditory-motor integration (42, 43). The early onset of auditorial processing abnormalities might further affect subsequent higher-order sensorial perception (44). In preschool children with ASD, alterations of FC of the Heschl gyrus was positively related to ASD symptoms (45). Moreover, reductions of gray matter volume of the Heschl gyrus were suggested to contribute to delays in spoken language (44).

Furthermore, we found that two FC links mediated the development of multiple RRBIs at different ages: between the left superior occipital lobe and right angular and between the left insula and left caudate. As a subregion of the striatum, the caudate was notably associated with RRBIs (46). Previous FC studies of the striatum found that RRBIs were associated with imbalanced corticostriatal connectivity, involving the striatum, frontal lobe, and parietal lobe (46–48). In the present study, FC between the left superior occipital lobe and right angular and between the left insula and left caudate decreased with age, which might contribute to the improvements of RRBIs. The angular gyrus was reported to be involved in semantic processing, word reading and comprehension, attention, and spatial cognition, and atypical structural and functional development of this region might contribute to autistic symptoms (30). As described in previous studies, alterations of FC of the angular gyrus were reported to be related to tactile disorders in ASD patients (49). Increases in volume of the angular gyrus were shown to be involved in motor impairments (30, 50). The insula and caudate were responsible for social cognitive and movement accordingly (15, 34–36). In the present study, we found that alterations of FC between the insula and caudate might further exacerbate impairments in social function and stereotyped behaviors.

The present study explored connectivity-based brain network features that supported RRBIs in ASD patients across development, but several limitations should be noted. First, this was a cross-sectional study with participants of different ages. The results should be cross-validated in a longitudinal analysis of subjects with follow-up. Second, the ASD patients mostly had high-functioning ASD. Future studies should also focus on low-functioning ASD patients. Third, we did not find FC links that were related to trajectories of self-injurious behaviors, compulsive behaviors, ritualistic behaviors, sameness behaviors, or restricted behaviors, which might be related to our limited sample size or cross-sectional study design. Thus, longitudinal studies with larger sample sizes are needed. Last but not the least, most participants in our study were boys, which might be a potential source of heterogeneity. Future studies with more girls are needed to test the generalizability of our findings.

In conclusion, the present study identified improvements in RRBIs in ASD patients with age, especially stereotyped behaviors, ritualistic behaviors, and restricted behaviors. The decrease in FC between the left superior occipital lobe and right angular and between the left insula and left caudate might contribute to improvements in multiple RRBIs in patients with ASD.

## Data Availability Statement

The datasets presented in this study can be found in online repositories. The names of the repository/repositories and accession number(s) can be found below: Data in present study was extracted from the open online database at http://fcon_1000.projects.nitrc.org/indi/abide/.

## Ethics Statement

Data in present study was extracted from the open online database. All participants and their caregivers have signed the contents at every sites. Written informed consent to participate in this study was provided by the participants' legal guardian/next of kin.

## Author Contributions

LLu, YB, and JL proposed the topic and main idea. AZ wrote the initial draft of manuscript with contributions from LLi, JL, and YB. AZ conducted the data analyses with help from LLi. JS, YB, JL, LLi, SC, LS, PL, and LLu critically revised the manuscript. All authors approved the results and final version of the manuscript.

## Funding

This study was supported by the National Natural Science Foundation of China (Nos. 81761128036, 81821092, and 82171514), Beijing Municipal Science and Technology Commission (Z181100001518005), the Fundamental Research Funds for the Central Universities (No. 71006Y2557), and PKU-Baidu Fund (No. 2020BD011). LLu was supported in part by the Postdoctoral Fellowship of Peking-Tsinghua Center for Life Sciences.

## Conflict of Interest

The authors declare that the research was conducted in the absence of any commercial or financial relationships that could be construed as a potential conflict of interest.

## Publisher's Note

All claims expressed in this article are solely those of the authors and do not necessarily represent those of their affiliated organizations, or those of the publisher, the editors and the reviewers. Any product that may be evaluated in this article, or claim that may be made by its manufacturer, is not guaranteed or endorsed by the publisher.
