# Supplementary material for: Connectivity-Based Brain Network Supports Restricted and Repetitive Behaviors in Autism Spectrum Disorder Across Development
Source: Front Psychiatry. 2022 Mar 25;13:874090. doi: 10.3389/fpsyt.2022.874090 (PMC8989843; doi:10.3389/fpsyt.2022.874090)
Supplement: Supplementary file 1 [file Data_Sheet_1.pdf]

Connectivity-based brain network supports restricted and repetitive behaviors in autism spectrum disorder across development

Anyi Zhang,[1] Lin Liu,[1, 2] Suhua Chang,[1] Le Shi,[1] Peng Li,[1] Jie Shi,[2] Lin Lu,[1, 3, *] Yanping Bao,[2, 4, *] Jiajia Liu,[5, *]

MATERIALS AND METHODS

Measures

ADOS and ADI-R Autism Diagnose Observation Schedule (ADOS) and Autism Diagnostic Interview-Revised (ADI-R) are widely used measurements, and recently accepted as the “gold standard” for diagnosing ASD (Schutte et al., 2018). These two measurements provide items assessing social function and restricted/repetitive behaviors in ASD. We extracted the subscales evaluating restricted/repetitive behaviors for every participants, and compared among different age-group.

Brain imaging processing

Scan parameters for each center The final sample included 147 ASD patients from nine research centers: BNI, IP, KKI, KUL, NYU, SDSU, TCD, and UCD. The detailed scan parameters were presented in the Table S1.

Results

As shown in **Table S2**, the RRBIs evaluated by ADOS significantly decreased after the childhood ($p = 0.017$), while the differences among younger adolescents, elder adolescents and adults were not profound, which was mostly consistent with the result using RBSR-6. However, the RRBIs assessed by ADI-R among four age groups were not significantly differ from each other. This result might be explained by the missing data on ADI-R in about half of the participants.

Table S1. The scan parameters of resting state fMRI at each center.

Data collection center	TR (ms)	TE (ms)	FOV (mm)	FA (°)	Resolution (mm^3)
BNI	3000	25	240	80	3.75×3.75×4.00
IP	2700	45	230	90	3.60×3.70×4.00
KKI	2500	30	288	60	3.00×3.00×3.00
KUL	2500	30	200	90	1.56×1.56×3.10
NYU_1	2000	15	240	90	3.00×3.00×4.00
NYU_2	2000	30	240	82	3.00×3.00×3.00
SDSU	2000	30	220	90	3.44×3.44×3.40
TCD	2000	27	240	90	3.00×3.00×3.20
UCD	2000	24	224	90	3.50×3.50×3.50

Note: TR, repetition time; TE, echo time; FOV: field of view; FA, flip angle; BNI, Barber National Institute; IP, Institut Pasteur; KKI, Kennedy Krieger Institute; KUL, Katholieke Universiteit Leuven; NYU, New York University; SDSU, San Diego State University; TCD, Trinity College Dublin; UCD, University College Dublin.

Table S2. Comparison of RRBIs evaluated by ADI-R and ADOS in different age groups (*n* = 147).

Questionnaire	(1) Children (*n* = 31)	(2) Younger adolescents (*n* = 52)	(3) Older adolescents (*n* = 33)	(4) Adults (*n* = 31)	*H*	*p*	*p* in *post hoc* test					
							(2)-(1)	(3)-(1)	(4)-(1)	(3)-(2)	(4)-(2)	(4)-(3)
ADI-R	6.00, 2.00	5.00, 3.00	5.00, 5.00	NA	4.31	0.230	NA	NA	NA	NA	NA	NA
ADOS	3.00, 3.00	2.50, 3.00	2.00, 3.00	2.00, 2.00	10.14	0.017	0.057	0.029	0.002	0.607	0.117	0.334

RRBIs, restricted and repetitive behaviors and interests; ADI-R, Autism Diagnostic Interview-Revised; ADOS, Autism Diagnose Observation Schedule; NA, not appliable. *Post hoc* tests were conducted to explore differences between age groups. In the *post hoc* test, (1) refers to children, (2) refers to younger adolescents, (3) refers to older adolescents, and (4) refers to adults.

References

Schutte, C., Richardson, W., Devlin, M., Hill, J., Ghossainy, M., and Hewitson, L. (2018). The relationship between social affect and restricted and repetitive behaviors measured on the ADOS-2 and maternal stress. *J Autism Dev Disord* 48(3), 751-758. doi: 10.1007/s10803-017-3453-1.
